# SELENBP1 inhibits progression of colorectal cancer by suppressing epithelial–mesenchymal transition

**DOI:** 10.1515/med-2022-0532

**Published:** 2022-09-01

**Authors:** Xiaotian Zhang, Runqi Hong, Lanxin Bei, Zhiqing Hu, Ximin Yang, Tao Song, Liang Chen, He Meng, Gengming Niu, Chongwei Ke

**Affiliations:** Department of General Surgery, Shanghai Fifth People’s Hospital, Fudan University, Shanghai, 200240, P.R. China; Department of Animal Science, School of Agriculture and Biology, Shanghai Jiao Tong University, Shanghai, 200240, China; Department of Radiology, Dongying New District Hospital, Dongying, Shandong Province, 257000, P.R. China; Department of General Surgery, Shanghai Fifth People’s Hospital, Fudan University, 801 Heqing Road, Minhang District, Shanghai, 200240, P.R. China

**Keywords:** selenium-binding protein 1, colorectal cancer, progression, tumorigenesis, epithelial–mesenchymal transition

## Abstract

Selenium-binding protein 1 (SELENBP1) is frequently dysregulated in various malignancies including colorectal cancer (CRC); however, its roles in progression of CRCs and the underlying mechanism remain to be elucidated. In this study, we compared the expression of SELENBP1 between CRCs and colorectal normal tissues (NTs), as well as between primary and metastatic CRCs; we determined the association between SELENBP1 expression and CRC patient prognoses; we conducted both *in vitro* and *in vivo* experiments to explore the functional roles of SELENBP1 in CRC progression; and we characterized the potential underlying mechanisms associated with SELENBP1 activities. We found that the expression of SELENBP1 was significantly and consistently decreased in CRCs than that in adjacent NTs, while significantly and frequently decreased in metastatic than primary CRCs. High expression of SELENBP1 was an independent predictor of favorable prognoses in CRC patients. Overexpression of SELENBP1 suppressed, while silencing of SELENBP1 promoted cell proliferation, migration and invasion, and *in vivo* tumorigenesis of CRC. Mechanically, SELENBP1 may suppress CRC progression by inhibiting the epithelial–mesenchymal transition.

## 1 Introduction

Colorectal cancer (CRC) is one of the most prevalent and fatal malignancies worldwide [1]. Radical surgery alone or in combination with adjuvant therapies has been effective in CRC patients at earlier stages; however, many of these patients experience recurrence within the next several years, while approximately 20% of CRC patients already have metastatic diseases at the time of diagnosis [2]. Although some contributing events have been identified for the progression of CRCs [3,4,5], our understanding of this process is still limited. Characterizing the underlying mechanisms of CRC progression and identifying novel biomarkers are therefore urgently needed.

Selenium-binding protein 1 (SELENBP1), one of the proteins that directly bind to selenium, is encoded by a gene located at 1q21.3 near the epidermal differentiation complex (EDC), which is closely related to terminal differentiation of the human epidermis [6]. Previous evidence showed that SELENBP1 participated in a variety of physiological processes, such as cell differentiation and maturation [7,8], protein transport and degradation [9,10], and H_2_S biosynthesis and adipogenesis [11], while mutations in SELENBP1 caused dysregulated methanethiol oxidation and extraoral halitosis [12]. As a binding partner for selenium, SELENBP1 may mediate the connection between selenium deficiency and carcinogenesis [13]. Actually, suppression of SELENBP1 has been associated with carcinogenesis and disease progression in CRC [7,14] and many other malignancies [15,16,17,18,19,20,21,22]; however, the underlying mechanism is not fully elucidated. Besides, the emerging open access datasets in recent years necessitate further validation of these pilot studies.

In the current study, we utilized data from the Human Protein Atlas (HPA), the Gene Expression Omnibus (GEO), and The Cancer Genome Atlas (TCGA) to determine SELENBP1 expression under physiological conditions and compare the expression of SELENBP1 between CRCs and colorectal normal tissues (NTs), as well as between primary and metastatic CRCs. We also used TCGA Colon Adenocarcinoma (COAD) and Rectum Adenocarcinoma (READ) datasets (combined into the TCGA cohort), and a tissue microarray cohort [the tissue microarray (TMA) cohort] to validate the association between SELENBP1 expression and CRC patient prognoses. Furthermore, we conducted both *in vitro* and *in vivo* experiments to explore the functional roles of SELENBP1 in CRC progression. Finally, we characterized the potential underlying mechanisms associated with SELENBP1 activities.

## 2 Materials and methods

### 2.1 Access to public datasets

HPA is an open access program that integrates various omics data to map all the human proteins in cells, tissues, and organs (www.proteinatlas.org) [23]. We used HPA to predict SELENBP1 expression under both physiological and pathological conditions. We then searched CRC datasets that compared gene transcription between normal colorectal mucosae and CRCs, or between primary and metastatic CRCs in the GEO database [24], as described in our previously report [25]. Eleven datasets were retrieved to compare SELENBP1 expression between NTs and CRCs, including GSE3629 [26], GSE28000 [27], GSE31279 [28], GSE37182 [29], GSE44861 [30], GSE87221 [31], GSE90627 [32], GSE106582 (unpublished data), GSE6988 [33], GSE21510 [34], and GSE62322 [35]. We also downloaded the TCGA COAD and READ datasets from UCSC Xena (https://xenabrowser.net/heatmap/) and combined them into one CRC dataset*The results <published or shown > here are in whole or part based upon data generated by the TCGA Research Network: http://cancergenome.nih.gov/.. These 12 datasets included 767 NTs and 1224 CRCs. In addition, 15 datasets containing both primary and metastatic CRCs were retrieved from GEO (GSE6988 [33], GSE18105 [36], GSE21510 [34], GSE27854 [37], GSE28722 [38], GSE29623 [39], GSE38832 [40], GSE40967 [41], GSE41568 [42], GSE51244 (unpublished data), GSE62322 [35], GSE71222 [43], GSE81582 [44], GSE81986 [45], and GSE68648 [46]), which included 1,534 primary and 667 metastatic CRCs.

### 2.2 Gene set enrichment analysis (GSEA)

To explore the potential mechanisms of SELENBP1 in CRC progression, a GSEA was employed using the combined TCGA COAD and READ datasets [47,48]. Gene sets with a false discovery rate *q*-value of <0.25 and a nominal *p* value of <0.05 were regarded as significantly enriched.

### 2.3 CRC TMA and immunohistochemical (IHC) staining

This study was approved by the Institutional Ethics Committee at Shanghai Fifth People’s Hospital and adhered to the principles listed in the Declaration of Helsinki. Informed consent was obtained from all patients. Collection of clinical samples and preparation of TMA were performed as described previously [25]. Detailed clinical variables of the TMA cohort, such as patient age and sex, are listed in Table 1. IHC staining and review of slides were performed as described in our previous report [49], using an immunoreactive score (IRS) system [50]. An anti-SELENBP1 rabbit polyclonal antibody was purchased from Sigma-Aldrich (HPA005741; St. Louis, MO, USA) and used at a dilution of 1:50.

**Table 1:** Clinical significance of SELENBP1 expression in colon cancers (*n* = 100)

Clinicopathological features	Cases (N)	SELENBP1 expression		
		Low	High	*P*-value
Sex				
Male	59	35	24	
Female	41	28	13	0.405
Age				
<67	43	26	17	
≥67	57	37	20	0.680
Histological grade				
G2	70	41	29	
G3	30	22	8	0.182
Tumor size (cm)				
<7	70	38	32	
≥7	30	25	5	**0.007**
Lymph node metastasis (n)				
<3	84	50	34	
≥3	16	13	3	0.157
pStage				
I/II	51	32	19	
III/IV	49	31	18	1.000
Gross typing				
Protruded	20	15	5	
Ulcerative	47	24	23	
Infiltrative	25	16	9	
Colloid	8	8	0	**0.030**
Location				
Transverse colon	7	5	2	
Left colon	42	26	16	
Right colon	51	32	19	0.904

### 2.4 Cell culture

A colon epithelial cell line fetal human cells (FHC) and four human CRC cell lines COLO205, COLO320DM, HCT116, and HT15 were obtained from the Cell Bank of Chinese Academy of Sciences (Shanghai, China). Cells were cultured in Dulbecco′s Modified Eagle′s Medium (DMEM) supplemented with 10% fetal bovine serum (FBS), 100 µg/mL of penicillin, and 100 mg/mL of streptomycin at 37°C with 5% CO_2_ in a humidified incubator (Thermo, Waltham, MA, USA) [25].

### 2.5 Cell viability assays

A Cell Counting Kit-8 (CCK-8) assay was conducted as described in our previously study [25]. Briefly, stably transfected HCT15 and HCT116 cells (5 × 10^3^ cells per well) were seeded in 96-well plates and cultivated overnight. Then, cells were serum-starved for another 24 h and 10% CCK-8 reagent (v/v in serum-free DMEM) was added to each well of the 96-well plates at 24, 48, 72, or 96 h. The absorbance at 450 nm was measured 1 h after addition of the reagent.

### 2.6 Cell proliferation assays

An EdU incorporation assay was performed using an EdU kit (C0071; Beyotime, Nantong, China) according to the manufacturer’s recommendation. Briefly, stably transfected HCT15 and HCT116 cells (1 × 10^5^ cells/mL) were seeded in 6-well plates and cultivated for 24–48 h, and then 10 μM/L EDU was added to cells. After 2 h, cells were fixed with 4% paraformaldehyde, permeabilized by 0.3% Triton X-100, and stained with the Click Additive Solution in the kit. Cell nuclei were stained with Hoechst 33342 for 10 min. The number of EdU-positive cells was counted under a microscope in five random fields. All assays were independently performed in triplicate.

### 2.7 Transwell migration and invasion assays

These assays were conducted as described in our previous report [51]. Briefly, cells (4 × 10^5^ cells/mL) were seeded in serum-free DMEM in the top chamber of a Transwell^®^ insert coated without (migration assay) or with (invasion assay) Matrigel. The medium containing 20% FBS in the lower chamber served as a chemoattractant. After incubation for 24 h at 37°C, the cells on the top side of the membrane were removed with a cotton swab and those on the bottom side were fixed with methanol for 20 min and then stained with crystal violet (0.1% in PBS) for 15 min. Five randomly selected fields per well were photographed, and the numbers of migrated cells were enumerated.

### 2.8 Protein extraction and western blotting (WB)

Proteins were extracted and plotted as previously described [51]. Primary and secondary antibodies used are listed in Table S1. Glyceraldehyde-3-phosphate dehydrogenase (GAPDH) (1:2,000 dilutions, rabbit anti-human; Beyotime Biotechnology, Shanghai, China) served as a loading control.

### 2.9 Ectopic expression or silencing of SELENBP1

Lentiviral plasmids expressing SELENBP1 (using GV367 vector), short hairpin RNA oligos of SELENBP1 (using GV248 vector), or respective controls were constructed by Shanghai Genechem Co., Ltd (Shanghai, China). The target sequences were CACTTATATGTATGGGACT (shSELENBP1) and TTCTCCGAACGTGTCACGT (scramble control). Transfection and construction of stable transfectants were performed as previously reported [25].

### 2.10 Animal experiments

Female athymic BALB/c nude mice of 6–8 weeks old were purchased from Charles River Laboratories (Beijing, China) and maintained in the Animal Experimental Facility of Normal University of Eastern China in a pathogen-free environment. HCT-15 cells (2 × 10^6^/mouse) stably expressing SELENBP1 or the vector were seeded subcutaneously into flanks of mice (*n* = 5 per group) and tumor growth was closely monitored twice a week (tumor volume = length × width^2^ × 3.14/6). One month after inoculation, tumors were isolated and weighed (g), and growth curves were drawn. Tumor samples were prepared for further use. All experiment procedures were conducted according to the Animal Care and Use guideline and were approved by the Animal Care Committee at the Normal University of Eastern China.

### 2.11 Immunofluorescence (IF) staining

The TMA was stained with antibodies against SELENBP1, E-cadherin, and N-cadherin by Wuhan Servicebio Technology Co., Ltd (Wuhan, China) according to their standard protocols as previously described [52] and signals were quantified by the same company using procedures recommended by Stephan et al. [53].

### 2.12 Statistical analyses

Analyses were performed using GraphPad Prism7 (GraphPad, San Diego, CA, USA), Microsoft Excel 2010 (Microsoft, Redmond, WA, USA), and SPSS statistical software for Windows, version 22 (SPSS, Chicago, IL, USA). Independent sample *t-*test or one-way analysis of variance was performed for comparisons of continuous variables. Nonparametric tests were performed if data did not follow a normal distribution. Pearson’s *χ* ^2^ test and Fisher’s exact test were used for categorical comparisons. IRSs of SELENBP1 staining in CRCs and paired NTs were compared using the Wilcoxon rank-sum test. Survival analyses were conducted using the Kaplan–Meier method and log-rank test. Univariate and multivariate survival analyses were conducted with a Cox proportional hazards regression model. Statistical significance was defined as a value of *p <* 0.05. All statistical tests were two-sided.


**Compliance with ethical standards:** The study protocol was approved by the Institutional Ethics Committee at the Fifth People’s Hospital of Shanghai, Fudan University (Ethical Approval Form no. 2017-097) and adhered to the principles of the Declaration of Helsinki. Written informed consent was obtained from each patient prior to tissue collection for experimentation.

## 3 Results

### 3.1 SELENBP1 expression was suppressed during CRC metastasis

**Figure 1:**
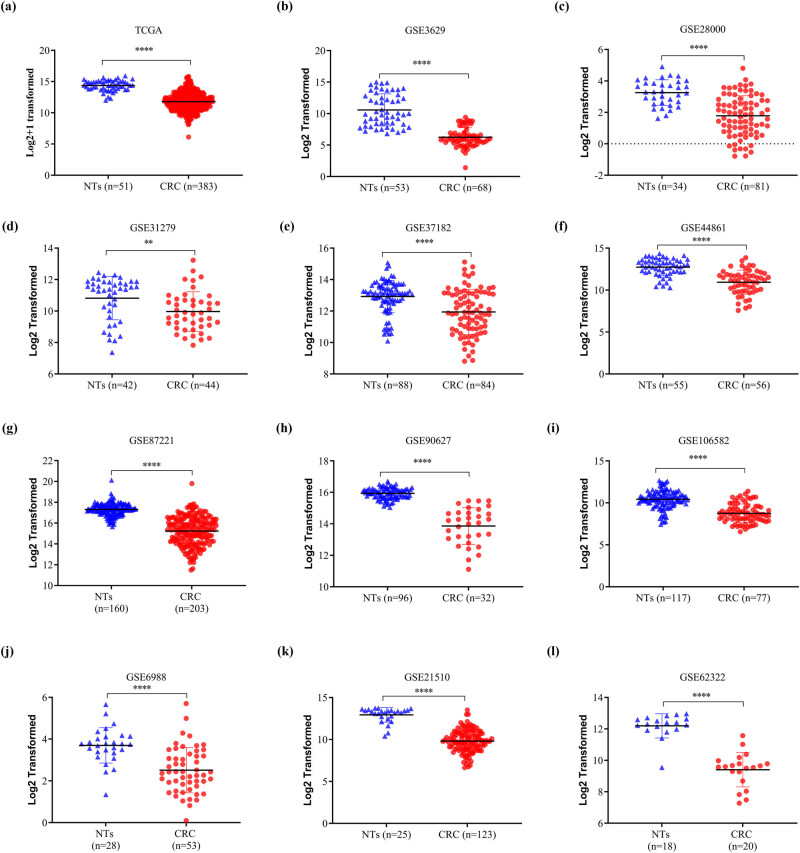
SELENBP1 expression is consistently downregulated in CRCs. The expression of SELENBP1 was compared between colorectal NTs and CRCs in 12 datasets from the TCGA and GEO databases (a–l). ^**^ *p* < 0.01; ^****^ *p* < 0.0001 vs the control group.

**Figure 2:**
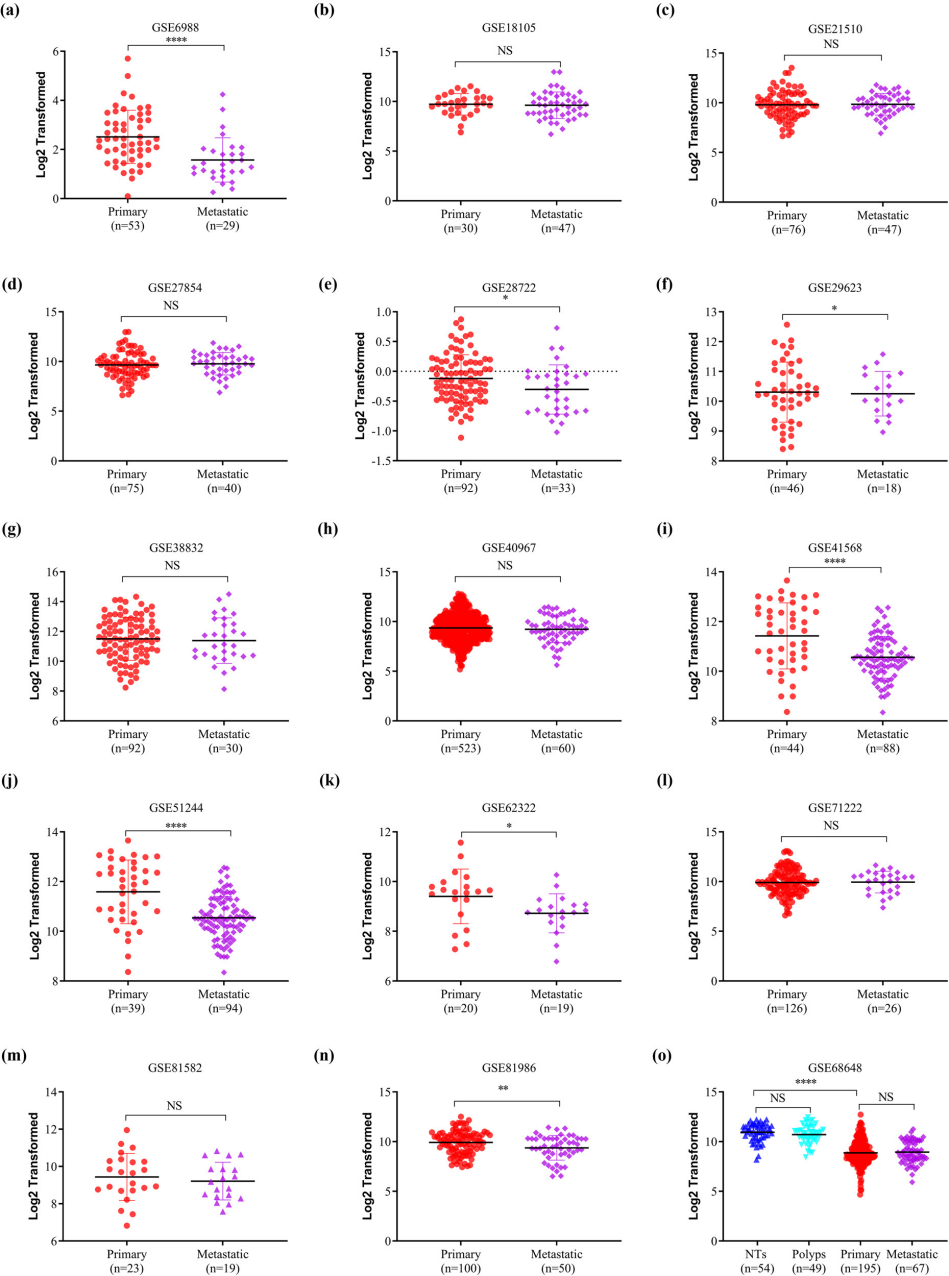
SELENBP1 expression is frequently downregulated in metastatic CRCs. The expression of SELENBP1 was compared between primary and metastatic CRCs in 15 datasets (a–o), and among different stages of colorectal tumors in one dataset (o). Abbreviation: NS, nonsignificant. ^*^ *p* < 0.05; ^**^ *p* < 0.01^; ****^ *p* < 0.0001 vs the control group.

**Figure 3:**
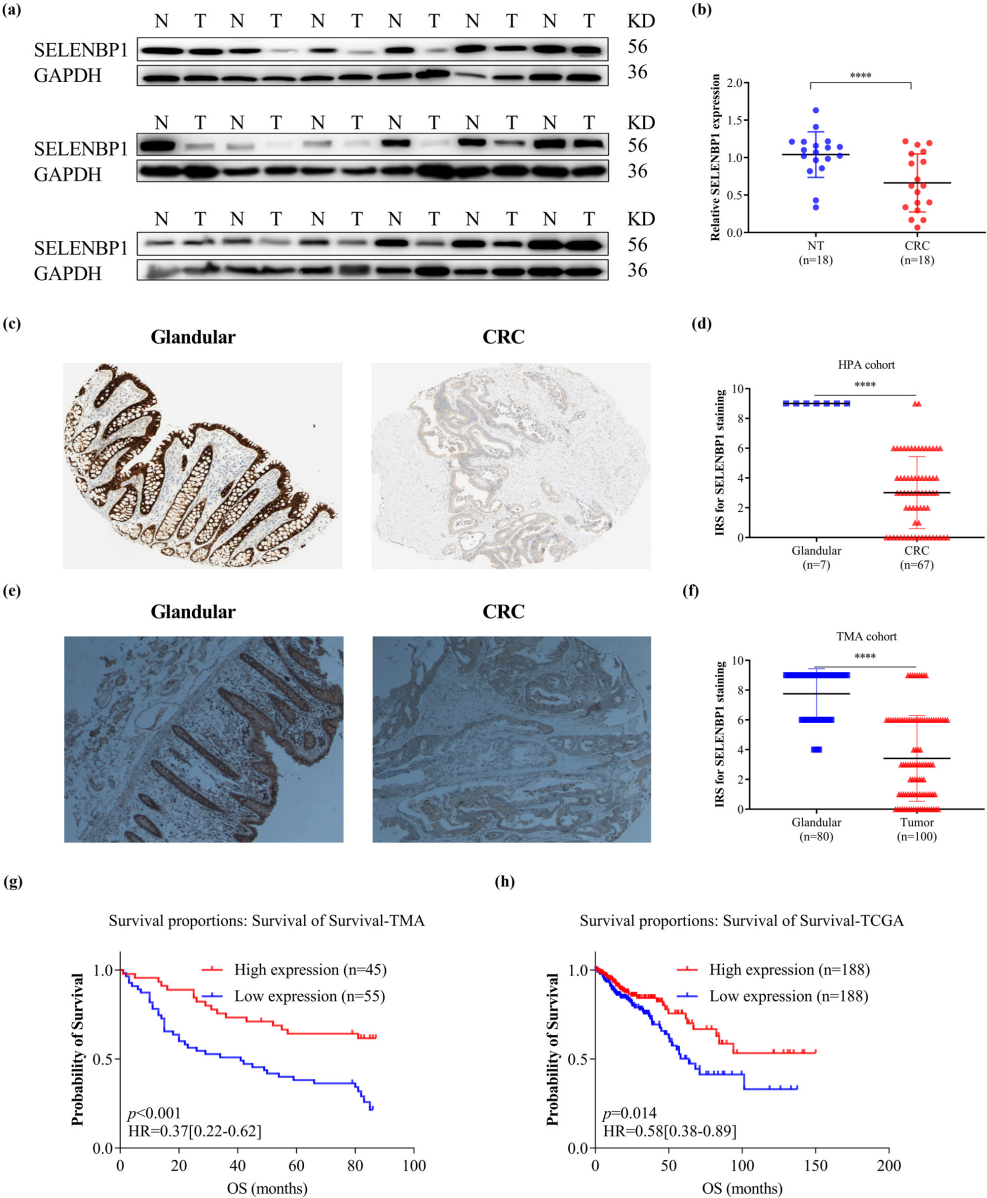
Suppressed expression of SELENBP1 in CRCs is associated with poor patient survival. The expression of SELENBP1 protein was detected by WB in 18 pairs of NTs and CRCs (a) and quantified by gray-scale analysis (b). Immunohistochemistry data of SELENBP1 were downloaded from the HPA database and compared between glandular tissues and CRCs using an IRS method (c and d). A TMA consisting of 100 CRCs and 80 NTs was stained with an anti-SELENBP1 antibody (d, 40×) and the IRS was evaluated (f). Kaplan–Meier plots were drawn for OS of patients in the TMA cohort (g) and TCGA cohort (h). Patients were stratified into low and high SELENBP1 expression groups according to *SELENBP1* mRNA expression in the TCGA cohort and IRS of SELENBP1 in the TMA cohort (<median vs ≥median). Values of *p* were obtained using the log-rank test. Censored data are indicated by the + symbol. ^***^ *p* < 0.001; ^****^ *p* < 0.0001 vs the control group.

The HPA database was used to examine SELENBP1 expression profiles under physiological conditions. As shown in Figure S1, the mRNA and protein expressions of SELENBP1 were most abundant in the colon, rectum, and thyroid, followed by liver, lung, and appendix, suggesting its functional relevance in these organs. To further elucidate the roles of SELENBP1 in CRC progression, 12 and 15 public datasets were used to examine the differences of SELENBP1 mRNA expressions between CRCs and colorectal NTs, and between primary and metastatic CRCs, respectively. The mRNA expression of SELENBP1 was dramatically decreased in CRCs compared to that in NTs (all, *p* < 0.01; Figure 1a–l). In addition, SELENBP1 expression was significantly lower in metastatic than in primary CRCs in seven out of 15 datasets (Figure 2a–o). Meanwhile, no significant difference in SELENBP1 expression was observed between NTs and polyps (Figure 2o). To validate these observations, we first examined the protein content of SELENBP1 in 18 paired CRC samples. As shown in Figure 3a and b, SELENBP1 expression was significantly decreased in most CRCs compared to their matched NTs. Then, IHC staining of SELENBP1 in colorectal NTs and CRCs in the HPA database indicated that SELENBP1 was distributed diffusively in the nuclei and cytoplasm and on the membrane, and its expression was significantly lower in tumor cells than in glandular cells (*p* < 0.0001; Figure 3c and d). These observations were further confirmed by IHC staining of SELENBP1 in 100 CRCs and 80 NTs, which showed that the intensity of SELENBP1 expression was much less in tumors than in adjacent NTs (*p* < 0.0001; Figure 3e and f). Taken together, these results suggest that suppression of SELENBP1 is common during carcinogenesis and frequent during the metastasis of CRCs.

### 3.2 Suppression of SELENBP1 in CRCs correlated with an unfavorable prognosis

To test whether SELENBP1 suppression in CRCs contributed to increased tumor invasiveness, we analyzed the relationships between SELENBP1 expression and clinicopathological variables. As shown in Table 1, SELENBP1 expression was significantly associated with tumor size and gross typing.

Next, we determined the relationship between SELENBP1 expression and patient outcomes in the tissue microarray cohort. Kaplan–Meier survival analysis revealed that patients with high SELENBP1 expression had a better overall survival (OS) than those with low expression (Figure 3g). Using multivariate analysis with a Cox proportional hazards model, high SELENBP1 expression was significantly associated with a better OS, after adjustment for age, tumor size, lymph node metastasis number, and TNM stage (Table 2). Similarly, a Kaplan–Meier survival analysis using the combined TCGA COAD and READ dataset also revealed that high SELENBP1 expression was correlated with a better OS in patients (Figure 3h). Along with those already reported in the literature [7,14], these findings clearly indicate that SELENBP1 is a prognostic marker in CRCs and its abundance in tumors could predict favorable prognoses.

**Table 2:** Univariate and multivariate Cox proportional hazard models for overall survival in CRC patients (*n* = 100)

Clinicopathological features	Univariate analysis		Multivariate analysis	
	HR [95% CIs]	*P*-value	HR [95% CIs]	*P*-value
Sex				
Male	1 [Reference]			
Female	0.78[0.44–1.38]	0.389		
Age				
<67	1 [Reference]		1 [Reference]	
≥67	1.90[1.05–3.44]	**0.033**	2.90[1.52–5.53]	**0.001**
Histological grade				
G2	1 [Reference]			
G3	1.40[0.78–2.50]	0.260		
Tumor size (cm)				
<7	1 [Reference]		1 [Reference]	
≥7	1.79[1.01–3.16]	**0.045**	1.64[0.90–3.01]	0.110
Lymph node metastasis (n)				
<3	1 [Reference]	**0.000**		
≥3	4.69[2.50–8.77]		4.30[1.99–9.31]	**0.000**
pStage				
I/II	1 [Reference]		1 [Reference]	0.068
III/IV	1.82[1.04–3.19]	**0.035**	1.92[0.95–3.87]	
Gross typing				
Protruded	1 [Reference]			
Ulcerative	0.80[0.39–1.65]	0.547		
Infiltrative	0.83[0.37–1.85]	0.650		
Colloid	1.23[0.43–3.54]	0.704		
Tumor location				
Left colon	1 [Reference]	0.821		
Right colon	0.94[0.53–1.66]			
Transverse colon	1.13[0.39–3.28]	0.822		
SELENBP1 expression				
Low	1 [Reference]		1 [Reference]	
High	0.42[0.23–0.76]	**0.004**	0.34[0.17–0.68]	**0.002**

CI, confidence interval; HR, hazard ratio.

### 3.3 SELENBP1 inhibited CRC cell proliferation, migration, and invasion

**Figure 4:**
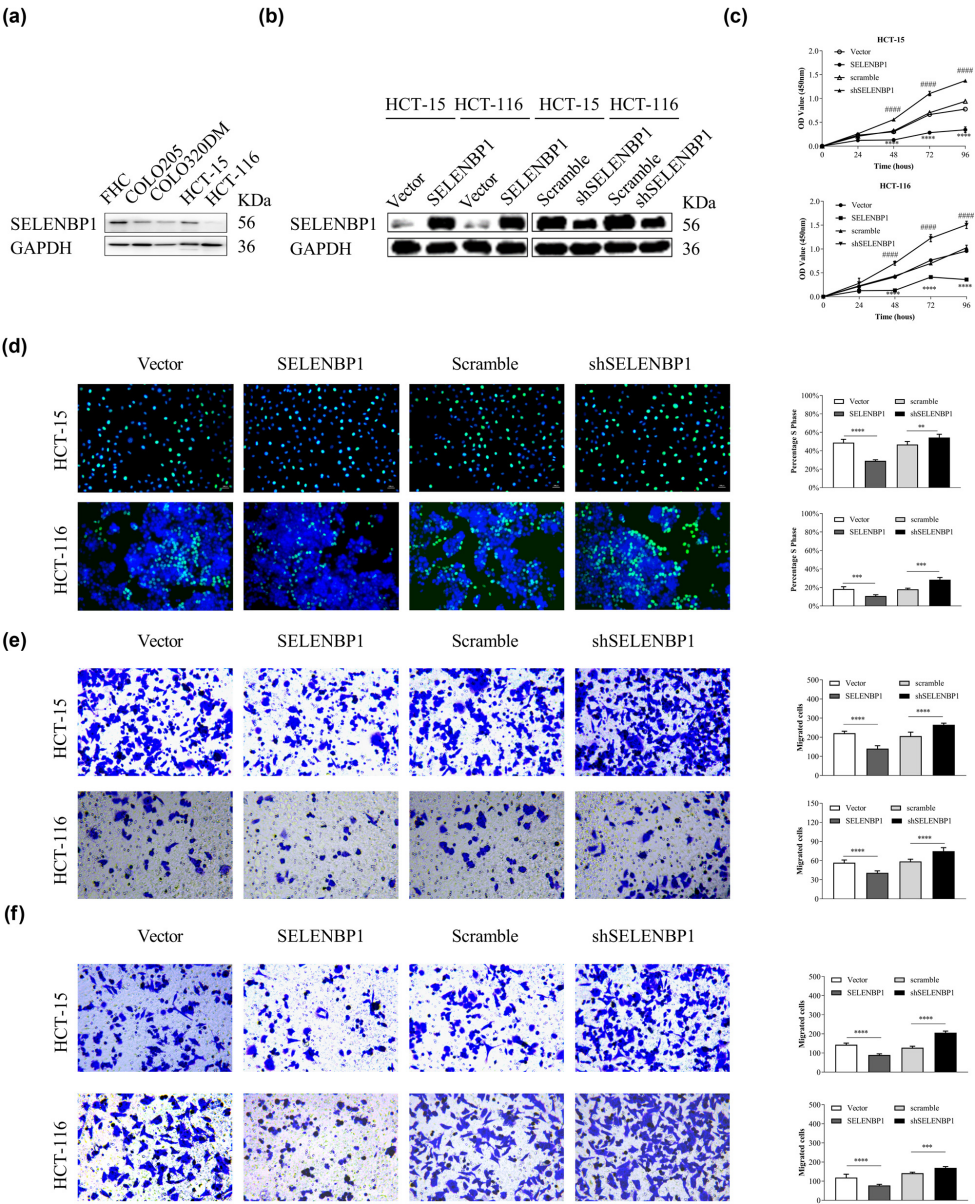
SELENBP1 inhibits cell proliferation, migration, and invasion of cultured CRC cells. The expression of SELENBP1 protein was determined in a fetal colon cell line FHC and several CRC cell lines (a). SELENBP1 was inducibly overexpressed and silenced in HCT-15 and HCT-116 cells (b). The *in vitro* effects of SELENBP1 on cell proliferation, migration, and invasion were evaluated by CCK-8 (c), Edu (d), Transwell migration (e), and invasion (f) assays, respectively. Experiments were repeated independently at least three times, and data are expressed as mean ± SEM (*n* = 3). *p < 0.05; ^**^ *p* < 0.01; ^***^ *p* < 0.001; ^****^ *p* < 0.0001 vs the control grou*p*; ^####^ *p* < 0.0001 vs the control group (for shSELENBP1 vs scramble in the CCK-8 assays only).

To investigate the *in vitro* activities of SELENBP1 in CRC, we first compared its expression in a fetal colon cell line FHC and four CRC cell lines. As shown in Figure 4a, the expression of SELENBP1 was decreased in CRC cell lines compared to that in FHC. We then induced or knocked down the expression of SELENBP1 in HCT-15 and HCT-116 cells using lentiviruses (Figure 4b) and carried out CCK-8, Edu, Transwell^®^ migration, and invasion assays. The results showed that overexpression of SELENBP1 inhibited while knocking down of SELENBP1 promoted cell viability (Figure 4c), proliferation (Figure 4d), migration (Figure 4e), and invasion (Figure 4f) in both cell lines. Taken together, these observations indicate that SELENBP1 has tumor-suppressive roles *in vitro*.

### 3.4 SELENBP1 may inhibit CRC progression by modulating epithelial–mesenchymal transition (EMT)

**Figure 5:**
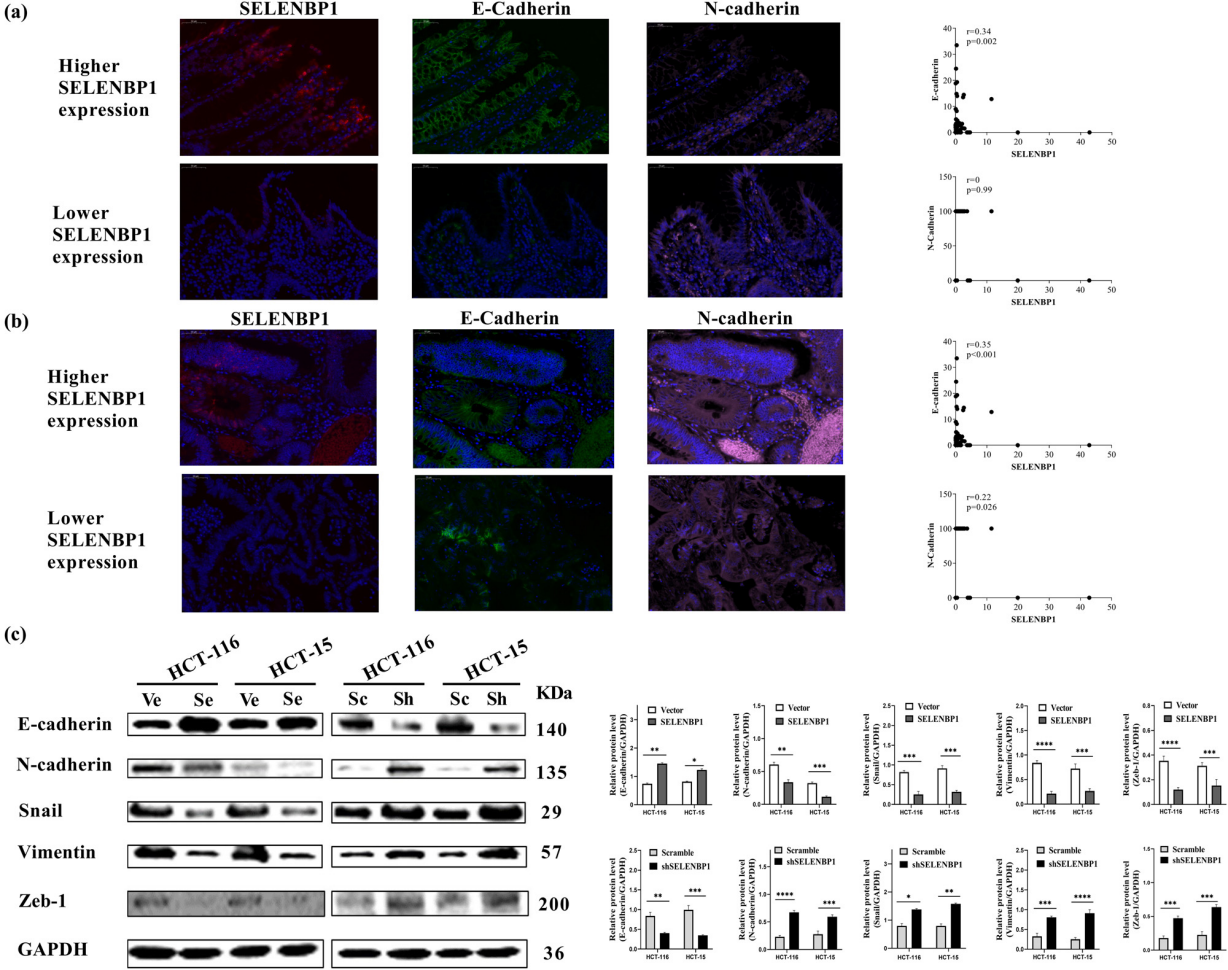
SELENBP1 inhibits EMT in CRC cells. A multicolor IF staining method was used to evaluate the expression and localization of SELENBP1 (red), E-cadherin (green), and N-cadherin (pink) in NTs (a) and CRCs (b), using the TMA cohort (scale bar = 50 μm). Percent of positive cells were calculated and correlation analyses were conducted based on the expression of these proteins (scatter plots on the right). Total proteins were extracted from HCT-15 and HCT-116 cells stably infected with SELENBP1, shSELENBP1, or relative control lentiviruses and were used to evaluate the expression of EMT markers and transcription factors by WB with GAPDH as a loading control (c). Experiments were repeated independently at least three times, and data are expressed as mean ± SEM (*n* = 3). *p < 0.05; **p < 0.01; ***p < 0.001; ****p < 0.0001 vs the control group.

To characterize the potential mechanism of SELENBP1 in inhibiting tumor progression, we first used the combined TCGA COAD and READ dataset to conduct a GSEA [54] and found that high SELENBP1 expression was negatively correlated with the hallmark EMT gene set (Figure S2a). Subsequent gene–gene correlation analyses using the same dataset further confirmed that the expression of SELENBP1 was positively correlated with that of CDH1 but negatively correlated with that of CDH2 and several other EMT markers and transcription factors both in NTs (Figure S2b) and CRCs (Figure S2c). To further confirm these observations, we investigated the relationship between SELENBP1 and E-cadherin or N-cadherin in NTs and CRCs by staining the tissue microarray with IF. As shown in Figure 5, SELENBP1 was located in colon mucosae and its expression correlated with that of E-cadherin in both NTs (A) and CRCs (B). By contrast, no consistent trend was observed between SELENBP1 and *N*-cadherin, as the expression of N-cad was diffusive in these samples. In addition, overexpression of SELENBP1 increased the expression of E-cadherin but decreased that of N-cadherin, SNAIL, Vimentin, and Zeb-1 in CRC cell lines, which was reversed in cells with SELENBP1 silencing (Figure 5c). Taken together, these results indicate that SELENBP1 played an active role in antagonizing CRC progression via modulating EMT.

### 3.5 SELENBP1 inhibited *in vivo* tumorigenesis

**Figure 6:**
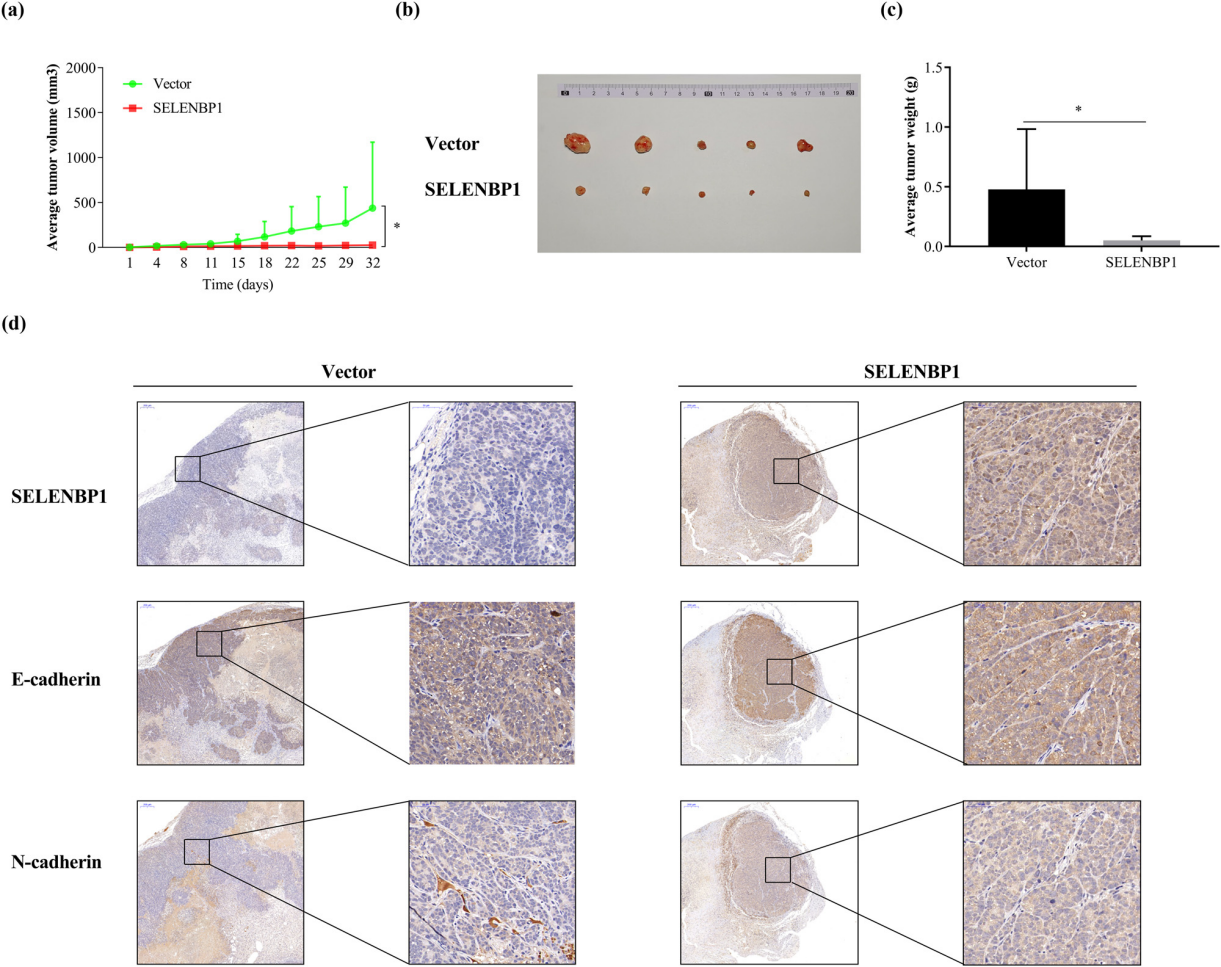
SELENBP1 inhibits tumorigenesis of CRC cells. HCT-15 cells stably overexpressing SELENBP1 or vector were inoculated subcutaneously into the right flank of nude mice (*n* = 5 per group). Tumor growth was monitored twice a week (a). On Day 32 after inoculation, tumors were removed, photographed (b), and weighed (c). Formalin-fixed and paraffin-embedded tumor blocks were cut into 5 μm sections and stained with respective antibodies against SELENBP1, E-cadherin, and N-cadherin (d). ^*^ *p* < 0.05 vs the control group.

To confirm whether SELENBP1 suppress CRC tumorigenesis *in vivo*, we inoculated HCT-15 cells that stably overexpressed SELENBP1 or the control subcutaneously into the flanks of nude mice (*n* = 5/group). As shown in Figure 6, SELENBP1 significantly inhibited tumor growth and tumor weight (a–c). Similar to the *in vitro* observations, SELENBP1 promoted E-cadherin but inhibited N-cadherin expression *in vivo* (Figure 6d).

## 4 Discussion

Although rapid progress has been made in recent years regarding the evolvement of CRCs, it is still incredibly challenging to interrupt this process. Identifying events that lead to progression of this malignancy could be beneficial, both clinically and scientifically. In the current study, we found that suppression of SELENBP1 might be such an event.

SELENBP1 was highly abundant in the colon and rectum under physiological conditions, but consistently suppressed in CRCs across different patient cohorts. A more remarkable suppression was observed in metastatic CRCs in some patient cohorts; in contrast, the expression of SELENBP1 was similar between NTs and polyps. Besides, suppression of SELENBP1 was correlated with increased tumor size and unfavorable patient prognosis, which validated the results from previous studies [7,14,55,56]. These observations, along with those from studies of other malignancies [15,16,17,22,57], suggest that suppression of SELENBP1 might be a common event during carcinogenesis across different malignancies, although the underlying mechanisms may vary.

Being a selenium-binding protein, SELENBP1 may duplicate some of the tumor-suppressive roles of selenium (Se), which is an essential trace mineral indispensable to human health [58]. In the form of selenocysteine, selenium constitutes the catalytic center of selenoproteins, such as glutathione peroxidases, iodothyronine deiodinases, and thioredoxin reductases. Many of these selenoproteins function as oxidoreductases that help maintain homeostasis of the internal environment by curbing the propagation of oxidative damages [59]. As such, selenium is regarded as an antioxidant, while inadequate selenium intake has been associated with increased cancer incidence and mortality [60]. Although initial clinical trials supported the use of dietary selenium replenishment in reducing both the incidence and mortality of cancer [61,62], later studies revealed that high selenium intake did not bring benefit, or even brought harmful effects [63,64,65]. The inconsistent efficacy of selenium as a candidate anticancer agent may in part be ascribed to its complex interactions with selenoproteins and selenium-binding proteins [9,13,17,55]. In the current study, we demonstrated that SELENBP1 has tumor-suppressive roles both *in vitro* and *in vivo*, in consistent with observations from other researchers [21,22,56]. Thus, the contribution of SELENBP1 should be considered in future selenium-oriented studies.

One intriguing observation was that SELENBP1 may inhibit EMT, which is one of the key processes mediating tumor metastasis [66]. The regulatory involvement of SELENBP1 in EMT has been reported in hepatobiliary tumors [22,67] but remains to be elucidated in CRC and other malignancies. Our investigation demonstrated that SELENBP1 induced the expression of E-cadherin and inhibited that of N-cadherin, which partly explains its suppressive roles during metastasis of CRC. The SELENBP1 gene located at chromosome 1q21.3 near the EDC, which contains genes that encode the S100A family members [6]. Amplification of 1q21.3, especially those fragments that encode the S100A family members, has been associated with tumor progression [68], while many of these family members are closely related to EMT and tumor metastasis [69–72]. Using the GEPIA database (http://gepia.cancer-pku.cn/), we found that the expression of SELENBP1 was negatively correlated with those of S100A1, S100A2, S100A3, S100A4, S100A7, S100A8, S100A9, S100A11, S100A12, and S100A13 in the TCGA COAD and READ datasets (data not shown). Thus, we surmise that SELENBP1 may interact with EDC genes to suppress EMT in CRCs.

Although this study presents some findings that are clinically and scientifically meaningful, there are some inherent limitations. First, we did not characterize the potential interaction of SELENBP1 with selenium and selenoproteins in CRC. Second, we did not observe a significant correlation between SELENBP1 expression and TNM staging in our patient cohort, maybe due to the sample size and patient heterogeneity. In addition, we only confirmed the *in vivo* tumor-suppressive activity of SELENBP1 using the subcutaneous xenograft model, since the cell lines we used failed to derive liver or lung metastasis. Finally, although we uncovered the inhibitory impact of SELENBP1 on EMT of CRCs, we did not further elaborate the underlying mechanism in the current study. These limitations should be addressed in future studies.

## 5 Conclusion

This study confirmed the active involvement of SELENBP1 in tumor progression of CRCs via modulating the EMT. SELENBP1 is therefore a candidate tumor suppressor, which should be further investigated in future studies.

## Abbreviations

CRC: colorectal cancer
EMT: epithelial–mesenchymal transition
GEO: Gene Expression Omnibus
GSEA: gene set enrichment analysis
HPA: Human Protein Atlas
IHC: immunohistochemical
SELENBP1: selenium-binding protein 1
TCGA: The Cancer Genome Atlas
TCGA-COAD: The Cancer Genome Atlas Colorectal Adenocarcinoma
TMA: tissue microarray

## Supplementary Material

Supplementary Material
