# Supplementary material for: SELENBP1 inhibits progression of colorectal cancer by suppressing epithelial–mesenchymal transition
Source: Open Med (Wars). 2022 Sep 1;17(1):1390–404. doi: 10.1515/med-2022-0532 (PMC9438969; doi:10.1515/med-2022-0532)
Supplement: Supplementary Material [file med-2022-0532-sm.pdf]

Supplementary material

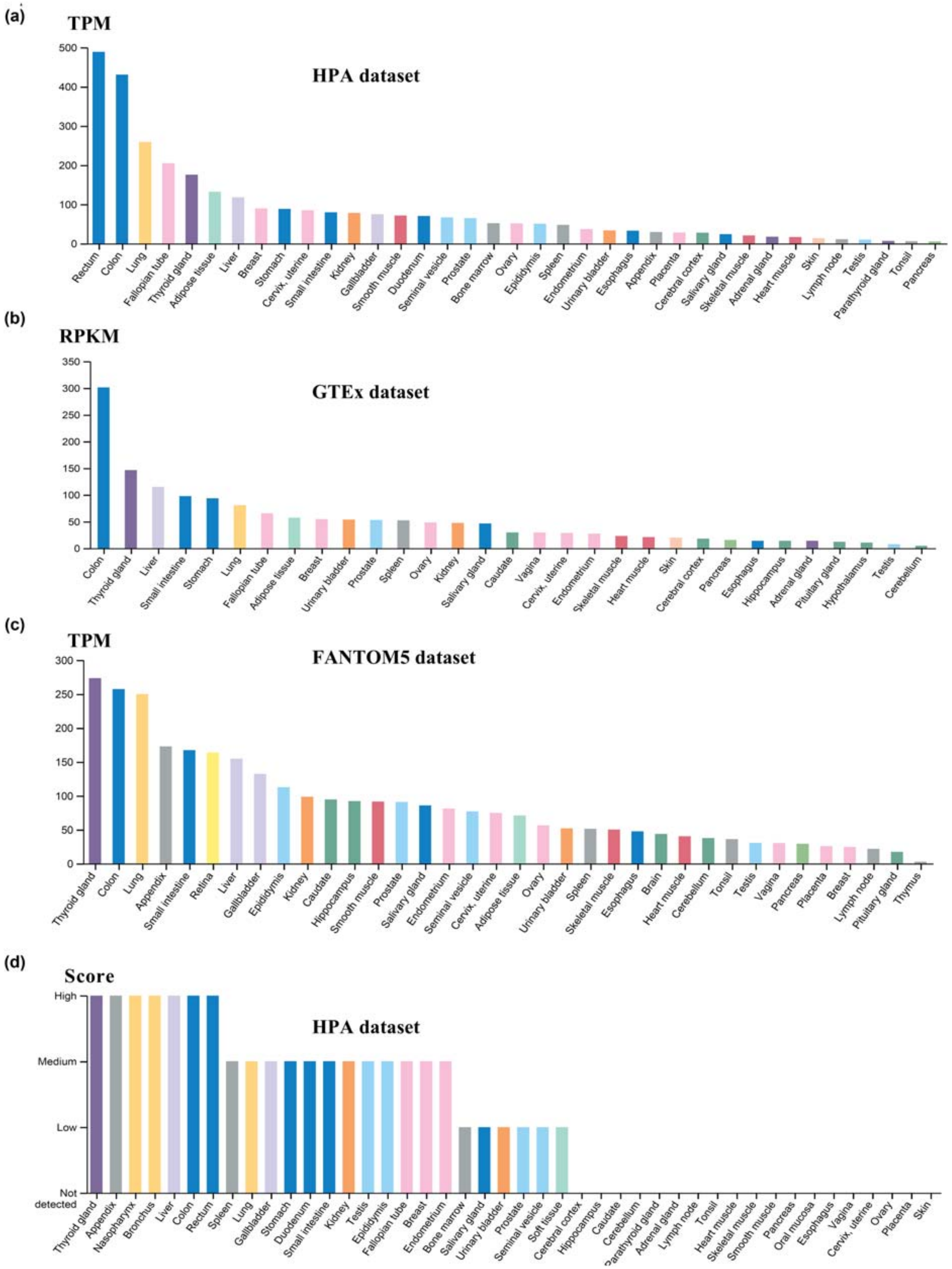

Figure S1: Expression of SELENBP1 mRNA and protein in various human organs under physiological conditions. SELENBP1 expression was predicted using the HPA database. A high abundance of SELENBP1 was observed in the colon, rectum, and thyroid in HPA, GTEx, and FANTOM5 datasets (a–d). Abbreviations: HPA, Human Protein Atlas; PRKM, Reads Per Kilobase Million; SELENBP1, selenium binding protein 1; TPM, Transcripts Per Kilobase Million.

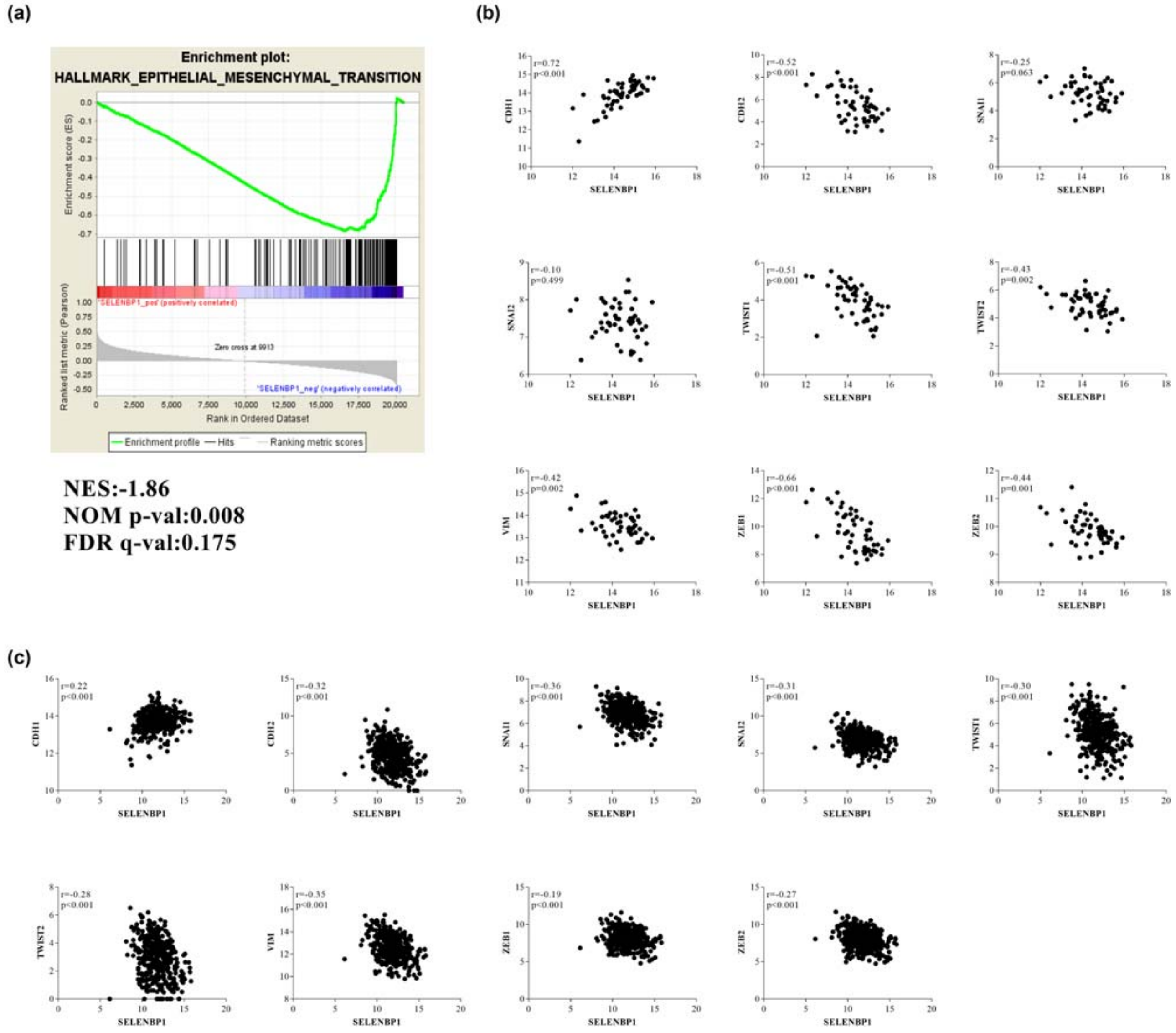

Figure S2: SELENBP1 is negatively associated with EMT in CRCs. GSEA enrichment plots using the combined TCGA COAD and READ datasets indicated that high expression of SELENBP1 was negatively correlated with the EMT gene signature (a). Gene-gene correlation analyses using the same datasets demonstrated that SELENBP1 was positively correlated with CDH1 and negatively correlated with CDH2, TWIST1, TWIST2, VIM, ZEB1, and ZEB2 in NTs (b); similarly, SELENBP1 was positively correlated with CDH1 and negatively correlated with CDH2, SNAI1, SNAI2, TWIST1, TWIST2, VIM, ZEB1, and ZEB2 in CRCs (c). Abbreviations: CDH1, cadherin 1; COAD, colon adenocarcinoma; CRC, colorectal cancer; EMT, epithelial-mesenchymal transition; FDR, false discovery rate; GSEA, gene set enrichment analysis; NES, normalized enrichment score; NT, normal tissue; READ, rectum adenocarcinoma; SELENBP1, selenium binding protein 1; SNAI1, Snail Family Transcriptional Repressor 1; TCGA, The Cancer Genome Atlas; TWIST1, Twist Family BHLH Transcription Factor 1; VIM, vimentin; ZEB1, Zinc finger E-box binding homeobox 1.

Table S1: Antibody information

Antibody name	Dilution	Category no.	Manufacturer
SELENBP1	1:1,000	ab90135	Abcam
E-cadherin	1:1,000	14472S	Cell signaling technology (CST)
N-cadherin	1:1,000	13116S	Cell signaling technology (CST)
Snail	1:1,000	3879S	Cell signaling technology (CST)
Vimentin	1:1,000	46173SF	Cell signaling technology (CST)
Zeb-1	1:500	70512S	Cell signaling technology (CST)
GAPDH	1:1,000	2118S	Cell signaling technology (CST)
Anti-mouse IgG, HRP-linked antibody	1:10,000	7076S	Cell signaling technology (CST)
Anti-rabit IgG, HRP-linked antibody	1:10,000	7074S	Cell signaling technology (CST)
